# Randomized, Double Blind, Placebo Controlled, Clinical Trial to Study Ashwagandha Administration in Participants Vaccinated Against COVID-19 on Safety, Immunogenicity, and Protection With COVID-19 Vaccine–A Study Protocol

**DOI:** 10.3389/fmed.2022.761655

**Published:** 2022-02-16

**Authors:** Arvind Chopra, Preeti Chavan-Gautam, Girish Tillu, Manjit Saluja, Swapnil Borse, Sanjeev Sarmukaddam, Susmita Chaudhuri, BCS Rao, Babita Yadav, Narayanam Srikanth, Bhushan Patwardhan

**Affiliations:** ^1^Center for Rheumatic Diseases, Pune, India; ^2^Center for Complementary and Integrative Health, Interdisciplinary School of Health Sciences, Savitribai Phule Pune University, Pune, India; ^3^Translational Health Science and Technology Institute (THSTI), Faridabad, India; ^4^Central Council for Research in Ayurvedic Science, New Delhi, India

**Keywords:** COVID-19, immunogenicity, *Withania somnifera*, vaccine, Ayurveda, Ashwagandha, immunoadjuvant

## Abstract

**Introduction:**

Vaccines have emerged as the most effective tool in the fight against COVID-19. Governments all over the world have rolled out the COVID-19 vaccine program for their populations. Oxford-AstraZeneca COVID-19 vaccine (COVISHIELD™) is widely used in India. A large number of Indian people have been consuming various traditional medicines in the hope of better protection against COVID-19 infection. Several studies have reported immunological benefits of *Withania somnifera* (Ashwagandha) and its potential as a vaccine adjuvant. We propose to study the safety, immunogenicity and clinical protection offered by a 6-month regimen of Ashwagandha in participants who volunteer to be vaccinated against COVID-19 (COVISHIELD^TM^) in the ongoing national program of vaccination.

**Methods and Analysis:**

We designed a prospective, randomized, double-blind, parallel-group, placebo-controlled, two-arm, exploratory study on healthy volunteers receiving the COVISHIELD^TM^ vaccine. The administration of Ashwagandha will begin within 7 days of the first or second dose of COVISHIELDTM. Primary outcome measure is immunogenicity as measured by SARS-CoV-2 spike (S1) and RBD-specific IgG antibody titres. Secondary outcome measures are safety, protective immune response and quality of life measures. All adverse events will be monitored at each time throughout the study. Participants will be tracked on a daily basis with a user-friendly mobile phone application. Following power calculation 600 participants will be recruited per arm to demonstrate superiority by a margin of 7% with 80% power. Study duration is 28 weeks with interim analysis at the end of 12 weeks.

**Ethics and Dissemination:**

Ethics approval was obtained through the Central and Institutional Ethics Committees. Participant recruitment commenced in December 2021. Results will be presented in conferences and published in preprints followed by peer-reviewed medical journals.

**Clinical Trial Registration:**

[www.ClinicalTrials.gov], identifier [CTRI/2021/06/034496].

## Introduction

Vaccines have emerged as the most effective tool in the fight against COVID-19. Governments all over the world have rolled out COVID-19 vaccine program for their populations. Clinical studies on vaccine efficacy suggest a role of high neutralizing antibody titers, robust CD8+ and Th1-biased CD4+ responses in protection against SARS-CoV-2 infection and also break through infections (1–4). With the emergence of new variants of concern, persistence of vaccine induced humoral and cell mediated immune response is being considered as key to fight against pandemic. There is ongoing global research on further enhancing the effectiveness of existing vaccines either by developing new vaccines against variants or by booster vaccination (5).

Botanical immunomodulators especially those based on traditional medicine offer opportunities for combined use with vaccines to induce optimum and persistent host immune responses (6). *Withania somnifera* (WS) also known as Ashwagandha in Ayurveda is a widely used medicinal plant for its immunomodulatory properties. Various studies on Ashwagandha have established its use as an immune-adjuvant in infection, inflammation and diseases such as arthritis and cancer (7). Immunoadjuvant activity of Ashwagandha has been reported in an infection model when co-administered with DPT (diptheria, pertussis, tetanus) vaccine (8). The long-term safety of Ashwagandha is well documented (9–13). It is also being investigated in comparison with hydroxychloroquine in a randomized controlled multi-centric clinical study for its prophylactic activity against COVID-19 in high-risk health care workers (CTRI/2020/08/027163, CTRI/2020/05/025332).

Based on our earlier studies, we hypothesize that co-administration of Ashwagandha with COVID-19 vaccine may result in better safety, immunogenicity and protection. Further, the vaccine efficacy, 22–90 days after a single dose of ChAdOx1 has been reported to be 76% (14). In this context it would be interesting to investigate whether Ashwagandha co-administration is able to provide a better immune response particularly after the first dose of the vaccine.

The primary hypothesis is that a long-term administration of Ashwagandha along with COVISHIELD^TM^ vaccine leads to a better protection against COVID-19 in healthy populations as compared to COVISHIELD^TM^ vaccine alone in terms of augmented immune-response, reduced incidence of breakthrough SARS-CoV-2 infections, good safety and tolerance.

We therefore propose to study the effect of Ashwagandha administration in participants vaccinated against COVID-19 vaccine (COVISHIELD^TM^) on safety, immunogenicity and protection in healthy population. COVISHIELD^TM^ consists of a replication-deficient chimpanzee adenoviral vector ChAdOx1, containing the SARS-CoV-2 structural surface glycoprotein antigen (spike protein; nCoV-19) gene (15, 16). Safety will be assessed by measuring the occurrence of adverse events. Immunogenicity will be assessed by measuring the SARS-CoV-2 S1 and RBD-specific IgG titers. Protective immune response will be evaluated by measuring SARS-CoV-2 specific (B1.617.2 and USA-WA1/2020 variants) neutralizing antibody titers; percent seroconversion after each vaccine dose; number of participants with SARS-CoV-2 breakthrough infections. An important aim of the study is also to evaluate the salubrious effects of Ashwagandha on physical and mental health and quality of life. Considering that long-term immune response to the vaccine will have implications for prevention of reinfection, it is also of interest to study the effect of Ashwagandha on the sustenance of the vaccine response. An auxiliary protocol is also planned to study the effect of Ashwagandha on T cell responses in vaccinated individuals. This study will help to understand the potential of Ashwagandha to improve vaccine response on a long-term basis.

## Methods and Analysis

### Patient and Public Involvement

This protocol was developed to address the immediate need to study safety and immunogenicity of Covid-19 vaccine among participants who orally consumed immunomodulating agents concurrently. Patients were not involved in the design, recruitment or conduct of the study. Results of the study will be disseminated to public after peer-review journal publication.

### Study Design

This will be a prospective, randomized, double blind, parallel group, placebo controlled, multi-centric study on healthy volunteers receiving the COVISHIELD^TM^ vaccine with a superiority design. The trial will be conducted at multiple locations as shown in the CTRI. Consenting and eligible volunteers will be enrolled and randomized to either of the two study arms (Ashwagandha plus COVISHEILD^TM^ vaccine or placebo plus COVISHIELD^TM^ vaccine) as per the protocol. The participants will be jointly evaluated by an Ayurvedic and a modern medicine physician for several clinical and laboratory measures. An Ayurveda based clinical assessment will also be carried out. The various assessments to be carried out are shown in Tables 1, 2.

**Table 1:** Clinical assessment schedule.

**Visit**	**Visit 0 Screening (Day −7 to −1)**	**Visit 1** **Enrollment and randomization (Day 1)**	**Visit 2 (4 wks)**	**Visit 3:(8 wks)**	**Visit 4 (12 wks)**		**Visit 5:(16 wks)**	**Visit 6** **Intervention completion1** **(24 wks)**	**Visit 7: Study completion (28 wks)**
Informed consent	X					**Interim analysis**			
Demographics	X								
Inclusion exclusion criteria	X								
Medical history	X								
Concomitant illness	X		X	X	X		X	X	X
Randomization		X							
Complete physical examination	X	X	X	X	X		X	X	X
WHO QOL BREF^@^		X			X			X	X
HR-BHF^#^		X			X			X	X
Vital signs	X	X	X	X	X		X	X	X
Ayurvedic evaluation: prakriti		X							
Ayurvedic evaluation: dhatu sarata		X	X	X	X		X	X	X
Ayurvedic evaluation:		X	X	X	X		X	X	X
Electrocardiogram	X							X	
Chest radiograph	X							X	
Study drug dispensation		X	X	X	X		X		
Adverse event assessment			X	X	X		X	X	X
Mobile App–Kavach–self reported questionnaire*		X	X	X	X		X	X	X
Completion and outcome report									X

* *To be completed daily by participants*.

# *HR-BHF: Health Related-Habits, Behavior and Fitness*.

@ *WHO QOL BREF: World Health Organization Quality of Life Instruments-short form*.

**Table 2:** Schedule of laboratory investigations.

**Visit**	**Visit 0 Screening (Day −7 to −1)**	**Visit 1 Enrollment and randomization (Day 1)**	**Visit 2 (4 wks)**	**Visit 3: (8 wks)**	**Visit 4 (12 wks)**	**Interim analysis**	**Visit 5:(16 wks)**	**Visit 6** **Intervention completion** **(24 wks)**	**Visit 7: Part 1 completion (28 wks)**
CBC: blood hemoglobin, total and differential count, platelets Erythrocyte sedimentation rate (Westergren)	X				X			X	
D-dimer	X				X			X	
LFT: serum liver enzymes-SGOT, SGPT, alkaline phosphatase Serum albumin, globulin, bilirubin	X				X			X	
RFT: blood urea nitrogen Serum creatinine	X				X			X	
Blood sugar profile (BSL fasting and HbA1c)	X				X			X	
Urine routine	X				X			X	
^+^Urine pregnancy test	X		X	X	X		X	X	X
^#^RT-PCR throat/nose swab testing for SARS-CoV2 RNA	X		X	X	X		X	X	X
SARS- CoV-2 S1, RBD and N Specific IgG		X	X	X	X		X	X	X
sVNT					X			X	X
SNT (Wuhan isolate USA-WA1/2020 and Delta variant B1.617.2)					X				X

# *RT-PCR throat nose swab test will be repeated in participants in case of development of any symptoms during the study duration*.

+ *for female participants*.

*Additional studies on measurement of secretory IgA and T cell responses are planned on a subset of the participants*.

If the vaccinated individual (after first or second dose) contracts COVID 19, then he/she shall be withdrawn from the study and will be treated under the supervision of COVID-19 physician till recovery. The participants who are withdrawn prematurely will be encouraged to continue follow up in a separate group as per the discretion and supervision of the study physician. The latter group will be permitted to use the current protocol methods and analyzed separately. All participants will be monitored for safety, immune response and occurrence of any breakthrough SARS-CoV-2 infection. An interim analysis at the end of 12 week is planned to evaluate the effect of Ashwagandha co-administration on safety and immune response following the first dose of the vaccine. The final analysis of data will be carried out after all the participants have completed the study at the end of 28 weeks.

### Recruitment, Screening, Consent

Apparently healthy volunteers (18–45 years) who have received either the first or second dose of the COVID-19 vaccine as part of the Government of India vaccination programme will be included in this study. The study will include up to 300 participants who have received the first dose and at least 900 participants who have received the second dose. The investigator will be responsible for obtaining written informed consent from each participant prior to screening as per the national ethics guidelines (17). An information sheet in language best understood by the participant, giving details of the study will be provided to the participant for study and discussion if any with the investigator. Enough time will be given to the participant to make a voluntary decision to participate. Participant will be encouraged to ask questions and clear all doubts in a face to face interview with the investigator. After fully satisfying with the various provisions of the research study the participant will be asked to sign the consent form. Participants will be screened comprehensively by the study physicians for eligibility and to exclude any contraindication to participation in the study. If found eligible, participants will be randomized and enrolled. Participants will be followed up at predetermined time endpoints (eight visits including screening and completion visits) as per protocol till study completion (week 28).

### Eligibility

#### Inclusion Criteria

Apparently healthy adults of either sex aged 18–45 years who have received either the first or second dose of COVID-19 vaccine; SARS-COV-2 RT-PCR negative volunteers; Written informed consent by the participants; Willing to comply with study protocol requirements; Female participants of childbearing potential with a negative urine pregnancy test.

#### Exclusion Criteria

Any known co-morbidity or acute illness with or without symptoms at the discretion of the investigator; History of known hypersensitivity to any component of the study material (Ashwagandha); Any confirmed or suspected condition with impaired/altered function of the immune system or conferring a possible risk suggested by investigations (e.g., increased levels of D-dimer); Women who are breast-feeding and pregnant; Individuals who are using any intervention (AYUSH or otherwise) for prophylaxis or for immune boosting or that may influence immune system e.g., steroids, methotrexate, anti-rheumatoid agents, drugs from AYUSH systems within 30 days prior to screening; Individuals who have received systemic immunoglobulins or blood products within 3 months prior to screening.

#### Withdrawal Criteria

The participants with drug compliance <70% (estimated by recorded tablet consumption), any serious adverse event, or those with positive RT-PCR tests, or clinical symptoms of COVID-19 will be withdrawn from the study. Withdrawn participants who are RT-PCR positive will be referred to a COVID-19 treatment facility for standard care.

#### Randomization

The participants eligible for enrolment will be randomized in a 1:1 ratio to Ashwagandha + COVISHIELD^TM^ or placebo + COVISHIELD^TM^ according to a pre-specified randomization scheme prepared by a statistician (SS) using a computerized system. Block randomization specific to each clinical study site will ensure equal number of participants per group with a small block size (e.g., a block of 4–6 participants). The statistician (SS) will design the randomization schedule following computer generated random numbers. The randomization scheme will consider the subsets of participants who are receiving first and second dose.

The patient number and the treatment allocation as per the schedule will be printed on a card, which will be sealed in an opaque envelope for each patient. The envelope of each patient will be delivered to respective trial sites. The treatment allocation will be concealed. Considering the study design, it is important to have a dosage form that ensures blinding. The tablets of Ashwagandha and placebo will have identical color and weight and will be dispensed in sealed bottles. The bottles will have same color and labeling. The bottles will be specific to the participant. The participant code will be written on each bottle containing the tablets.

#### Monitoring

Following randomization at baseline, participants will be physically checked by study physicians at all visits till the completion of study. At all the time points, assessment of participants will also include in-depth inquiry of possible symptoms of COVID-19 in family members and close associates. The participants will be provided with a user friendly mobile app (named COVID Kavach) designed for COVID-19 studies for daily tracking and monitoring of onset of fever/ respiratory and any other symptoms/vaccine related side effects (18). The daily response of the participant will be seen by the site physician and study co-ordinators; anonymous participant data which will only be identified by a unique study ID known to the site investigator will be saved in a dedicated central study server. In addition, a telephonic contact will be made by the site coordinator with the participant once in every 2 weeks till study completion to find out about any matter concerning health or the study. The schedule of enrolment, interventions, and assessments is provided in Table 3.

**Table 3:** Schedule of enrolment, interventions, and assessments.

	**Study period**							
	**Enrolment**	**Allocation**	**Post-allocation**					**Close-out**
**Timepoint**	***D***_**−07to −01**_	***D***_**01**_	***W***_**04**_	***W***_**08**_	***W***_**12**_	***W***_**16**_	***W***_**24**_	***W***_**28**_
**Enrolment**:								
Eligibility screen	X							
Informed consent	X							
Allocation		X						
Interventions*:								
[Daily dosing of placebo or Ashwagandha]		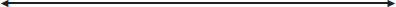						
**Assessments**:								
Concomitant illness	X	X	X	X	X	X	X	X
Randomization		X						
Complete physical examination	X	X	X	X	X	X	X	X
WHO QOL BREF, HR-BHF		X			X		X	X
Vital signs	X	X	X	X	X	X	X	X
Ayurvedic phenotype: prakriti		X						
Ayurvedic assessment: dhatu sarata, srotasa pariksha		X	X	X	X	X	X	X
ECG, Chest-X ray	X						X	
Study drug dispensation,		X		X		X		
Compliance			X	X	X	X	X	X
Mobile App–Kavach–self reported questionnaire		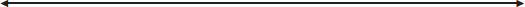						
Blood hemoglobin, total and differential count, platelets, ESR, SGOT, SGPT, alkaline phosphatase, serum albumin, globulin, bilirubin, blood urea nitrogen, serum creatinine,	X				X		X	
D-dimer	X				X		X	
HbA1c, blood sugar fasting, urine routine	X				X		X	
Urine pregnancy test	X		X	X	X	X	X	X
RT-PCR throat/nose swab testing for SARS-CoV2 RNA	X		X	X	X	X	X	X
SARS- CoV-2 S1, RBD and N Specific IgG		X	X	X	X	X	X	X
sVNT					X		X	X
SNT (variants-USA-WA1/2020 and Lineage B.1.617.2; delta variant)					X			X
Completion and outcome report								X
AE	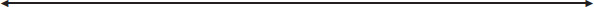							

*Additional studies on measurement of secretory IgA and T cell responses are planned on a subset of the participants*.

#### Compliance

The participants will be daily monitored for fever, respiratory and any other symptoms and vaccine related side effects. Telephonic follow up will be done once in every 2 weeks throughout the study. The study coordinator will contact the participants for monitoring symptoms and ensure compliance. Medication logs will be maintained for both vaccine use and Ashwagandha tablet consumption. On each return visit, the participant will be instructed to bring all unused medication and a tablet count will be performed and recorded in the medication log. COVID-Kavach App and regular telephonic contact will be used to remind participants to adhere to study medication instructions.

#### Trial Intervention

The study includes two arms as Ashwagandha and Placebo The participants from placebo arm will have immunogenic effects only of vaccine, whereas participants in Ashwagandha arm may have additional safety, immunogenicity and protection from disease. Thus, the placebo arm will serve as a control to ascertain adjuvant effects offered by Ashwagandha.

Arm 1: Ashwagandha tablets (*n* = 600): Dose of Ashwagandha prescribed for this study is one tablet of the standardized aqueous extract equivalent to 4 gm Ashwagandha root powder per day administered within 7 days of the first or second dose of vaccine and continued up to 24 weeks. Each tablet used in this trial shall contain aqueous extract equivalent to 4 grams Ashwagandha root powder.

Arm 2: Placebo (*n* = 600): One placebo tablet will be administered daily beginning within 7 days of the first or second vaccine dose and continued up to 24 weeks. The appearance and weight of placebo tablet (containing microcrystalline cellulose) will be same as that of Ashwagandha tablet.

One tablet of Ashwagandha or placebo will be administered daily (on empty stomach in the morning) with water. Quality of Ashwagandha roots is ensured in accordance with prescribed pharmacopeia standards (19).

A window period of 7 days will be allowed on either side of the scheduled visit for exceptional or any other anticipated reasons at the discretion of the investigator.

#### Clinical Outcomes

##### Primary Outcome Measure

Immunogenicity as measured by SARS-CoV-2 spike (S1) and RBD-specific IgG antibody titres.

##### Secondary Outcome Measures

Safety as measured by occurrence of adverse events. Protective immune response as measured by SARS-CoV-2 specific neutralizing antibody titer; Duration of sustenance of SARS-CoV-2 virus neutralizing antibody titer; percent seroconversion after each vaccine dose; number of participants with SARS-CoV-2 breakthrough infections; proportion of participants with COVID 19 (RT PCR assay confirmed) after the first dose and both showing evidence of infection before vaccination (Time frame: Day 1 vaccination to Week 12 or second dose and whichever is earlier); proportion of participants with COVID-19 (RT-PCR assay confirmed) after completion of the second dose (Time frame: Second dose to week 28 or study completion) and Quality of life measured using standard WHO Quality Of Life-BREF 1997 (20) and recently designed and validated Health Related-Behavior Habit Fitness (CRD Pune version 2020) questionnaires. All clinical and laboratory investigations and assessments pertaining to general health, and to rule out concurrent medical illness including COVID-19 will be carried out as per Tables 1, 2.

#### Adverse Events

All adverse events (AEs) and adverse events following immunization (AEFI) occurring during the study will be recorded and monitored as per ICH-GCP (2016) (21) and ICMR guidelines (17). Safety would also be assessed in case of all withdrawals. All adverse events will be recorded and meticulously followed till resolution. Serious adverse events will require immediate reporting by the study investigator to the Sponsor and Ethics Committee, within 24 h, of becoming aware of the SAE. All Adverse Events will be classified by the study physician and recorded in the AE form. Any symptoms recorded on COVID-Kavach App will be immediately followed by investigators to rule out COVID-19. The Data Safety Monitoring Board (DSMB) will be formed to monitor safety of the participants and assess safety data. Clinical assessments and lab investigations will be carried out as per Tables 1, 2.

#### Concomitant Medication

As a general principle, concomitant medication of any kind will be kept to a minimum and will necessarily require clearance from a primary care physician and with full information provided to the study physician. All concomitant medication will be recorded in the study case record form (CRF) by the study physician. All complete CRFs/eCRF duly signed by the investigator and counter signed by principal investigator will be submitted within 2 weeks of the completion of the study to the Chief Clinical Coordinator. Although the study includes healthy participants, the medication required for any new illness during the study will be taken under supervision of the primary care physician and meticulously recorded in the participant CRF. Participants will be permitted general symptomatic medications like paracetamol and non-steroidal anti-inflammatory drugs for short period (<4 days) and including for any untoward mild to moderate symptoms like headache and body aches following vaccination. Patients will be carefully explained not to consume any other herbal and home remedies. Patients will be advised not to use any kind of food and nutrition supplement. Similarly, participants will be advised not to consume any kind of vitamins and/or minerals without a specific advice of physician.

### Statistical Analysis

#### Sample Size and Power

The sample size is calculated based on the primary objective of improved immunogenicity of the vaccine with administration of Ashwagandha. Also, it was felt important to obtain a reasonably suitable (non-probabilistic) sample size for safety and tolerability. As there was no prior data on the expected effect size of the difference between the two study groups, a superiority margin of 7% was decided by expert consensus between the biostatistician (SS) and senior authors (AC, GT, PCG, and BP). The sample size was calculated with 80% power and after consideration of interim analysis at the end of 12 weeks; assuming vaccine efficacy of 80% (22). The calculated sample size with the provision of one interim analysis was rounded to 600 subjects per study arms and totalling 1,200. The study will include upto 300 participants who have received the first dose and at least 900 participants who have received the second dose.

An interim analysis is planned after completing 12 weeks of intervention. The sample size of the study is decided based on the plan of one interim analysis. The sample size for interim analysis will be minimum 300 (with alpha = 2.96%).

#### Analysis

A standard statistical software package will be used for statistical tests (parametric and non-parametric). The analysis will be both intention-to-treat (ITT) and as per-protocol (PP). All randomized participants will be included in the safety analysis. All statistical processing will be performed using standard statistical package (SPSS, BMDP, and CIA) unless otherwise stated. The data will be individually analyzed for central tendencies (mean, median), range, standard deviation and 95% confidence intervals for each intervention arm in the study. Titer data will be “log” transformed before any analyses [and GMT, GSD, etc. will be reported and will be used for testing with respect to that parameter. Data will be tabulated and graphically shown using standard format using MS Excel and other software programs. Standard parametric (Student *t*-tests–paired and unpaired, ANOVA, etc.) and non-parametric statistical tests (Wilcoxon Signed Rank test, Mann-Whitney “U” test, Kruskal-Wallis test, etc.) will be used depending upon the normality of the data, level of measurement and appropriateness/applicability. Fisher's exact test or Chi square will be used to test the proportion of participants classified as efficacy failures and those with AE. All AEs will be presented as proportion and with Wilson's recommended exact binomial 95% Confidence Interval.

#### Electronic Database

An e-database will be created to capture all the important variables required for the final study report and supervised by the CRO. Access to e-database will be limited to the site investigator (only for the particular site), study co-ordinators and project team and CRO, and will be password protected. The CRO will be responsible for the security and back up of the study e-database. The database will be checked for missing data and inconsistencies by the CRO from time to time in close liaison with the site investigator. The CRO will lock the database after informing the clinical coordinator and sponsor and then submit copy for statistical analysis and report writing both for interim and final report.

#### Trial Care and Record Retention

The participants will be advised to continue the usual protective measures like physical distancing, respiratory and hand hygiene, and wearing masks. They will be advised to follow guidelines of Ministry of AYUSH for COVID-19 prophylaxis. All clinical study documents will be retained by the investigator for at least 5 years after the study and await instructions of their final disposal from the sponsor. All protocol amendment will be approved by the respective Ethics Committees before implementation. In the event of an emergency of any type, medical or otherwise, the site investigator will inform the appropriate Ethics Committee, CRO, study coordinators and sponsor within 24 h. The Sponsor (CCRAS) will provide insurance coverage with respect to any liability caused by the investigational products (Ashwagandha) and or study related procedures in connection with the clinical study. The terms and conditions will apply as specified in the insurance policy document. The quantum of compensation for the Serious Adverse Events leading to the death will be given to the study participant/patients' nominee as per recommendations of the EC, DSMB, and as per the regulatory guidelines.

## Discussion

Several vaccines have been approved in the first year of the pandemic on priority for an emergency use. We still do not have a clinically acceptable specific drug to treat COVID-19. The case fatality rate in India still hovers around 2% in the second pandemic year. It is indeed challenging to predict disease progression at an early stage. Agreeably, vaccination is reassuring, however the future remains somewhat uncertain. The success with vaccines is influenced by several host and environmental factors. The data on safety, tolerance and efficacy is not adequate. In case of natural COVID-19 infections it has been observed that the titers of IgG against spike protein may last for about 6 months, while the immunological memory may last up to 8 months (23, 24). However, the duration of protection after vaccination has not been established so far. A looming fear of more waves may sweep away some of the benefits of the vaccination in the population. The world indeed is immensely stressed and mental health is at stake (25). How to improve the performance of the vaccines remains a pivotal question. The current study protocol is planned and designed against this perspective.

Previous clinical studies have highlighted the role of humoral as well as cell-mediated immune responses in recovery from SARS-CoV-2 infection (26, 27). In previous studies the selective-Th-1 upregulating activity of Ashwagandha was demonstrated and in infection models, co-administration of Ashwagandha with DPT vaccine resulted in improved antibody titres against lethal challenge of pertussis toxins (8). *In silico* network ethnopharmacological investigations also suggest the ability of bioactive compounds of Ashwagandha to modulate immune pathways involved in innate and adaptive immune responses (28, 29). Further, the interim efficacy data for ChAdOx1 nCoV-19, evaluated in four trials across UK, Brazil and South Africa shows a protection of 64.1% after one standard dose (14). COVISHIELD^TM^ has been administered in two doses to a large population in India. We hypothesize that co-administration of Ashwagandha with the COVISHIELD^TM^ vaccine may enhance the antibody titres particularly after the first dose of the vaccine. A sustained long-term immune response to the vaccine has implications for prevention of reinfection. Therefore, the effect of Ashwagandha on the sustenance of neutralizing antibody titres up to 4 months after the second dose will also be evaluated in this study (30). Ashwagandha is also known to improve overall mental wellbeing.

Currently, the study involves the assessment of neutralizing antibodies against two well-characterized variants of concern (B1.617.2 and USA-WA1/2020 variants). Recently, a new variant B.1.1.529, named Omicron has emerged and it is likely that other newer variants may emerge during the course of the study. It would be interesting to evaluate the responses to these variants. As and when fully characterized BEI resources are available for new variants of concern, the investigators will take the decision to modify and make the necessary amendments to the protocol. Serum samples are being preserved at all time points of the study and may be used for such additional studies.

Our trial has the potential to have a significant impact on current vaccination program by improving immunogenicity, protection and general wellbeing with simple, safe and affordable intervention. In this trial we expect a beneficial effect beginning soon after the first dose of vaccination. We hope to investigate biological plausibility that the immune protection offered by a long-term co-administration of Ashwagandha may effectively address some of the vexing issues associated with the vaccination.

## Strengths and Limitations of this Study

- Novel randomized placebo-controlled study of COVID-19 vaccine and immune adjuvant co-administration on safety, immunogenicity, and protection to reduce breakthrough infections.
- State of art immune assays to measure specific antibodies to SARS-CoV-2 to demonstrate both persistent and late upsurge in immune response.
- Daily tracking of participants using a study-specific designed mobile app.
- Participants may be reluctant to donate blood repeatedly for immune assays; compliance with the test drug may be a challenge.

## Ethics Statement

Ethical approval was obtained through the Central Ethics Committees of CCRAS New Delhi and respective institutional Ethics Committees. Participant recruitment commenced in December 2021. The patients/participants provided their written informed consent to participate in this study.

## Author Contributions

BP and AC contributed to conceptualization. AC, GT, PCG, SB, NS, and BP contributed to methodology and study plan and design. SS contributed to sample size and statistics. SC contributed to immunological component. MS, BR, and BY contributed to structuring, review, registration, and coordination. BP, AC, and GT prepared first draft, attended reviewers comments, and revision to make a final version. All authors have made substantial contributions in the development of the study protocol.

## Funding

This study was funded by Central Council for Research in Ayurvedic Sciences (CCRAS) and Ministry of AYUSH, Government of India, New Delhi (Grant No. 802/2021-22).

## Conflict of Interest

BR, BY, and NS work at CCRAS, MoA, Government of India (GOI), New Delhi. AC is chief clinical coordinator designated by MoA. BP is Chairman Interdisciplinary AYUSH R and D Task Force on COVID-19 established by MoA. The remaining authors declare that the research was conducted in the absence of any commercial or financial relationships that could be construed as a potential conflict of interest.

## Publisher's Note

All claims expressed in this article are solely those of the authors and do not necessarily represent those of their affiliated organizations, or those of the publisher, the editors and the reviewers. Any product that may be evaluated in this article, or claim that may be made by its manufacturer, is not guaranteed or endorsed by the publisher.
