# Corrigendum: Randomized, Double Blind, Placebo Controlled, Clinical Trial to Study Ashwagandha Administration in Participants Vaccinated Against COVID-19 on Safety, Immunogenicity, and Protection With COVID-19 Vaccine–A Study Protocol

**DOI:** 10.3389/fmed.2022.888928

**Published:** 2022-03-29

**Authors:** Arvind Chopra, Preeti Chavan-Gautam, Girish Tillu, Manjit Saluja, Swapnil Borse, Sanjeev Sarmukaddam, Susmita Chaudhuri, BCS Rao, Babita Yadav, Narayanam Srikanth, Bhushan Patwardhan

**Affiliations:** ^1^Center for Rheumatic Diseases, Pune, India; ^2^Center for Complementary and Integrative Health, Interdisciplinary School of Health Sciences, Savitribai Phule Pune University, Pune, India; ^3^Translational Health Science and Technology Institute (THSTI), Faridabad, India; ^4^Central Council for Research in Ayurvedic Science, New Delhi, India

**Keywords:** COVID-19, immunogenicity, *Withania somnifera*, vaccine, Ayurveda, Ashwagandha, immunoadjuvant

In the original article, there was an error in the sentence “One tablet of Ashwagandha or placebo will be administered twice daily (on empty stomach in the morning and after dinner) with water.”

The correction has been made to the **Eligibility** section, “*Trial Intervention*” sub section and the corrected sentence appears below:

“One tablet of Ashwagandha or placebo will be administered daily (on empty stomach in the morning) with water.”

The authors apologize for this error and state that this does not change the scientific conclusions of the article in any way. The original article has been updated.

## Publisher's Note

All claims expressed in this article are solely those of the authors and do not necessarily represent those of their affiliated organizations, or those of the publisher, the editors and the reviewers. Any product that may be evaluated in this article, or claim that may be made by its manufacturer, is not guaranteed or endorsed by the publisher.

